
JOURNAL INFORMATION
==============================
NLM Title Abbreviation: ScientificWorldJournal
Journal ID: ScientificWorldJournal
NLM Journal ID: 101131163
Journal ID: TSWJ

The Scientific World Journal

ISSN: 2356-6140
EISSN: 1537-744X
Publisher: Wiley

ARTICLE INFORMATION
==============================
PMCID: PMC6083888
DOI: 10.1100/tsw.2001.415
Article ID: 279603
Article version: 1
Subjects: Extended Abstract

Capsaicin-Mediated Neurogenic Vasodilatation in Neurokinin-1, NK1, Receptor Knockout Mice

Grant A. D.
Brain S. D.
Centre for Cardiovascular Biology and Medicine, New Hunt’s House, Guy’s Campus, King’s College, London, SE1 1UL, UK
Publication date: 2001
Electronic publication date: 2001 Aug 10
Volume: 1
First page: 26
Last page: 26
Copyright: Copyright © 2001 A. D. Grant and S. D. Brain.
Copyright year: 2001
License: This is an open access article distributed under the Creative Commons Attribution License, which permits unrestricted use, distribution, and reproduction in any medium, provided the original work is properly cited.
License URL: https://creativecommons.org/licenses/by/3.0/

==============================
4th International Meeting on Calcitonin Gene-Related Peptide (CGRP)
*TheScientificWorld* (2001) 1(S1), 26
ISSN 1532-2246; DOI 10.1100/tsw.2001.415

### CAPSAICIN-MEDIATED NEUROGENIC VASODILATATION IN NEUROKININ-1, $NK_1$, RECEPTOR KNOCKOUT MICE

A.D. Grant and S.D. Brain
Centre for Cardiovascular Biology and Medicine, New Hunt's House, Guy's Campus, King's College, London, SE1 1UL, U.K.

Capsaicin induces tachykinin $NK_1$ receptor-dependent neurogenic oedema in the mouse ear and this model has been used to determine the absence of $NK_1$ receptors in $NK_1$ knockout ($NK_1$-/-) mice[1]. We have now simultaneously measured neurogenic vasodilatation and oedema in the ear.

Wild-type and $NK_1$-/- Sv129+C57BL/6 mice were used in this study. Anaesthesia (urethane, 25% w/v; 100ml/10g) was induced. Oedema was assessed by the accumulation of $^{125}$I-albumin, and blood flow by laser Doppler flowmetry. Responses were measured for 1 h after topical application of capsaicin to one ear and vehicle to the contralateral ear. Capsaicin induced oedema in wild-type Sv129+C57BL/6 mice ($p < 0.001$), but not in $NK_1$-/- mice, as expected[2]. However, neurogenic vasodilatation ($p < 0.05$) was observed in both wild-type and $NK_1$-/- mice, and remained in wild-type mice in the presence of the $NK_1$ antagonist SR140333 (480 nmol/kg). Interestingly, increased blood flow in the $NK_1$-/- mice was significantly ($p < 0.05$) greater than that in the wild-type mice. It was substantially blocked in $NK_1$-/- mice pretreated with the CGRP antagonist $CGRP_{8-37}$ (400nmol/kg), or wild-type mice treated with both SR140333 and $CGRP_{8-37}$, but not in wild-type mice treated with $CGRP_{8-37}$ alone.

The results suggest that neurogenic vasodilatation is a consequence of CGRP and $NK_1$ receptor mediated responses in the mouse. They also indicate that some form of interaction between functional CGRP and $NK_1$ receptors may occur.

A.D. Grant is supported by a BHF studentship.

### REFERENCES

1. Bozic, C., Lu, B., Hopken, U.E., et al. (1996) Science 273, 1722–1725.
2. Cao, T., Gerard, N.P., and Brain, S.D. (1999) Am. J. Physiol. 277, 476–481.
